# Nuclear Norm Clustering: a promising alternative method for clustering tasks

**DOI:** 10.1038/s41598-018-29246-4

**Published:** 2018-07-18

**Authors:** Yi Wang, Yi Li, Chunhong Qiao, Xiaoyu Liu, Meng Hao, Yin Yao Shugart, Momiao Xiong, Li Jin

**Affiliations:** 1 Ministry of Education Key Laboratory of Contemporary Anthropology, Department of Anthropology and Human Genetics, School of Life Sciences, Fudan University, Shanghai, China; 2 State Key Laboratory of Genetic Engineering, Collaborative Innovation Center for Genetics and Development, School of Life Sciences, Fudan University, Shanghai, China; 3 Unit on Statistical Genomics, Division of Intramural Division Programs, National, Institute of Mental Health, National Institutes of Health, Bethesda, MD USA; 4 Human Genetics Center, School of Public Health, University of Texas Houston Health Sciences Center, Houston, Texas USA; 5 Six Industrial Research Institute, Fudan University, Shanghai, China; 6 Human Phenome Institute, Fudan University, Shanghai, China

## Abstract

Clustering techniques are widely used in many applications. The goal of clustering is to identify patterns or groups of similar objects within a dataset of interest. However, many cluster methods are neither robust nor sensitive to noises and outliers in real data. In this paper, we present Nuclear Norm Clustering (NNC, available at https://sourceforge.net/projects/nnc/), an algorithm that can be used in various fields as a promising alternative to the k-means clustering method. The NNC algorithm requires users to provide a data matrix M and a desired number of cluster K. We employed simulated annealing techniques to choose an optimal label vector that minimizes nuclear norm of the pooled within cluster residual matrix. To evaluate the performance of the NNC algorithm, we compared the performance of both 15 public datasets and 2 genome-wide association studies (GWAS) on psoriasis, comparing our method with other classic methods. The results indicate that NNC method has a competitive performance in terms of F-score on 15 benchmarked public datasets and 2 psoriasis GWAS datasets. So NNC is a promising alternative method for clustering tasks.

## Introduction

Clustering is defined as grouping objects in sets. A good clustering method will generate clusters with a high intra-class similarity and a low inter-class similarity^1^. There are several classic and representative clustering methods which are widely used in biological data analysis, including k-means clustering^2,3^, Partitioning Around Medoids (PAM)^4^, hierarchical clustering (Hcluster)^5^, Clustering Large Applications (CLARA)^4^, Agglomerative Nesting (AGNES)^4,6,7^, Divisive Analysis Clustering (DIANA)^4^, Clusterdp^8,9^ and DBSCAN^10^.

K-means clustering is a popular method of vector quantization in data mining. The term “k-means” was first used by MacQueen^2^ in 1967 and the standard algorithm was first proposed by Lloyd^3^ in 1957. K-means clustering is typically used to partition n observations into k clusters in which each observation belongs to the cluster with the nearest mean, serving as a prototype of the cluster.

The Partitioning Around Medoids (PAM) is a clustering algorithm related to the k-means clustering and the medoids shift algorithm^4^. Both the k-means and PAM are partitional (breaking the dataset up into groups) and both attempt to minimize the distance between points labeled to be in a cluster and a point designated as the center of that cluster. In contrast to the k-means clustering, PAM chooses data points as centers and works with a generalization of the Manhattan Norm to define distance between data points. The PAM method was proposed in 1987 and is a classical partitioning technique of clustering that clusters the dataset of n objects into k clusters.

Hierarchical clustering (Hcluster)^5^ is a method of cluster analysis which seeks to build a hierarchy of clusters. Strategies for hierarchical clustering generally fall into two subcategories: agglomerative and divisive^1^. In general, the merges and splits can be achieved in a greedy manner. The results of hierarchical clustering are usually presented in a dendrogram.

Clustering large applications (CLARA)^4^ is characterized by taking a small portion of the data as a sample without considering the entire data set. It extracts multiple sample sets from the data set and uses the best cluster as output, by using PAM for each sample set. CLARA can handle a larger data set than PAM. Agglomerative nesting (AGNES)^4,6,7^ algorithm belongs to hierarchical clustering method. AGNES initially takes each object as a cluster, afterwards the clusters are merged step by step according to certain criteria, using a single-link method. The level of similarity of the two clusters is measured by the similarity of the nearest pair of data points in the two different clusters. The clustering process is repeated until all objects finally meet the number of clusters. The DIANA (Divisive analysis)^4^ algorithm is a typical split clustering method. DIANA first places all objects in a cluster and then subdivides them into smaller clusters until the desired number of clusters is obtained. Density-based methods include Clusterdp^8,9^, DBSCAN^10^, etc. Clusterdp^8,9^ is a recently developed method based on the idea that centroids are characterized by a higher local density than their neighbors and by a comparably high distance from objects with higher density.

Obviously, each clustering method has its own strengths and drawbacks. Although some methods work well on one data set, it may give poor results on another data set. The K-means clustering algorithm is compromised when feature is highly correlated and is extremely sensitive to outliers, because its distance measurement can be easily influenced by extreme values, and it is also computationally difficult (NP-hard)^11–15^. The most time-consuming part of PAM is the calculation of the distances between objects. CLARA relies on the sampling approach to handle large datasets^4^, therefore, the quality of CLARA’s clustering results depends greatly on the size of the sample. AGNES algorithm does not undo what was previously carried out. No objective function is directly minimized. Sometimes it is difficult to identify the correct number of clusters by using the dendrogram. DIANA chooses the object with the maximum average dissimilarity and then moves all objects to this cluster that are more similar to the new cluster than to the remainder.

We consider that the objective of clustering is to minimize the “residuals” within clusters. We can use norms to measure “residuals”, like L2~L0 norms^16^. For example, L2 error is the square error, L1 error is the nuclear norm and L0 error is the rank of the residual matrix. Minimizing nuclear norm not only reduces the quantitative error (variance) but also reduces the qualitative errors (rank) and encourages the residuals to be embedded in low dimensional spaces. To achieve this goal, we developed the Nuclear Norm Clustering (NNC) method (available at https://sourceforge.net/projects/nnc/), a highly accurate and robust algorithm used for clustering analysis. Nuclear Norm Clustering aims to improve the accuracy of clustering. In this paper, we compared the performance of NNC with that of other seven methods, using 15 publically available datasets. We then tested the performance of NNC on two psoriasis genome-wide association study (GWAS) datasets^17–20^.

## Methods

To apply our method to a specific dataset, users need to provide a data matrix M and the desired number of cluster K. The objective function to minimize is the nuclear norm of the pooled within class residual. The nuclear norm of a matrix is defined as the sum of singular values of the matrix.

Suppose we had a candidate class label vector A, where A[i] was an integer indicating that the i_th_ sample belong to the A[i]_th_ cluster. We first calculated the means/center of each class. Then for each sample/row, we subtracted its corresponding class mean, forming a pooled residual matrix. Then we performed singular value decomposition (SVD)^21^ to obtain nuclear norm. This procedure could be denoted as NN(A).

We used simulated annealing^22^ to choose an optimal A that minimize NN(A). First we initially random guess some A. Then we randomly change one sample’s label obtain A′, and test if it improves the nuclear norm. If Uniform(0, 1) < exp((NN-NN’)/T) then A = A′, where T is the annealing parameter. The algorithm is shown in Table 1.

**Table 1:** The pseudocode of Nuclear Norm Clustering.

subroutine **Nuclear Norm (A, M)** {Parameter: assignment vector A, normalized data matrix M. 1: Calculate the cluster center/mean C using A and M 2: For each rows i in M, subtract the corresponding cluster mean vector C_Ai_. 3: Perform SVD of the matrix M = USV^T^ return the sum of singular values}
**Nuclear Norm Clustering of normalized data matrix M** 1: randomly assign the assignment vector A 2: NN = Nuclear Norm (A, M) 3: repeat N iterations 4: {A′ = A 5: A′ [random sample] = random cluster 6: NN′ = Nuclear Norm (A′) 7: T = N/(100*(iter + 1.0)) 8: if (Uniform (0, 1) < exp ((NN-NN′)/T)) 9: {A = A′ 10: NN = NN′}} 11: A is the clustering result

## Bechmarking

We benchmarked eight methods: k-means clustering, Partitioning Around Medoids (PAM), Hierarchical clustering (Hcluster, using Euclidean metric to calculate dissimilarities), Clustering Large Applications (CLARA), Agglomerative Nesting (AGNES), Divisive Analysis Clustering (DIANA), Clusterdp (Clusterdp was chosen as the representative of density-based methods) and Nuclear Norm Clustering (NNC). We used the NNC software available at https://sourceforge.net/projects/nnc/ and implemented the other seven methods using various R packages: factoextra^23^ and densityClust^24^. To evaluate the performance of benchmarked clustering methods, we used the macro-averaged F-score^25,26^. Benchmarking was performed on a desktop PC equipped with an Intel Core i7-4790 CPU and 32 GB of memory. The parameters tested were shown in Supplemental Materials 1, 2 and 4.

### Benchmarking Public Datasets Study

Overall 15 public datasets were included: *spambase*^27^, *Indian liver patient*^28^, *blood transfusion service center*^29^, *pima Indians diabetes*^30^, *parkinsons*^31^, *QSAR biodegradation*^32^, *Ionosphere*^27^, *pathbased*^33^, *mammographic mass*^34^, *breast cancer wisconsin diagnostic*^35^, *seeds*^36^, *wine*^27^, *jain*^37^, *flame*^38^, *iris*^27^.

### Applications on GWAS Dataset Study

We applied each of the aforementioned method to two psoriasis genome-wide association (GWAS) genetic datasets^17–20^. We obtained the dataset, a part of the Collaborative Association Study of Psoriasis (CASP), from the Genetic Association Information Network (GAIN) database, a partnership of the Foundation for the National Institutes of Health. The data were available at http://dbgap.ncbi.nlm.nih.gov. through dbGap accession number phs000019.v1.p1. All genotypes were filtered by checking for data quality^18^. We included 1590 subjects (915 cases, 675 controls) in the general research use (GRU) group and 1133 subjects (431 cases and 702 controls) in the autoimmune disease only (ADO) group. A dermatologist diagnosed all psoriasis cases. Each participant’s DNA was genotyped with the Perlegen 500 K array. Both cases and controls agreed to sign the consent contract, and controls (≥18 years old) had no confounding factors relative to a known diagnosis of psoriasis.

In our previous work^18^, we found that when the number of SNPs as predictors was chosen as 50, the independent ADO (testing) dataset could reach the maximum AUC^39^ (AUC = 0.7063) using logistic regression prediction model. Thus we used SNP ranking methods, considering allelic association p-values (on the Psoriasis GWAS dataset of GRU group), to select top 50 associated SNPs (take 5 intervals, such as 5, 10 …, 50, shown in Supplementary Materials 4) and then compared the performance of different clustering methods on two Psoriasis GWAS datasets (both GRU and ADO group).

## Results

### Results from public datasets

Table 2 summarizes the macro-averaged F-score of all methods on 15 public datasets. NNC, together with Clusterdp and Hcluster, all performed best in 4 datasets. PAM performed optimally in 2 datasets. Following PAM, DIANA performed best only one datasets. Furthermore, we observed that the datasets in which NNC performed better were linearly separable (especially in *iris, seeds* and *wine* datasets).

**Table 2:** Macro-averaged F-score of all methods on 15 datasets.

Datasets	sample	feature	class	k-means	PAM	Hcluster	CLARA	AGNES	DIANA	Clusterdp	NNC
*spambase*	4601	57	2	0.4756	0.7594	0.8257	0.3771	0.3779	0.3779	0.6088	**0.8492**
*Indian liver patient*	579	10	2	0.4122	0.5406	**0.6196**	0.5418	0.4163	0.4122	0.5981	0.5837
*blood transfusion service center*	748	4	2	0.5630	0.5710	**0.6482**	0.5849	0.4658	0.5547	0.6304	0.5554
*pima Indians diabetes*	768	8	2	0.5803	0.6202	0.6918	0.6169	0.4131	0.6385	0.6100	**0.7079**
*parkinsons*	195	22	2	0.4682	0.6748	0.7013	0.6733	0.4231	0.4073	**0.7529**	0.6376
*QSAR biodegradation*	1055	41	2	0.5025	0.7112	0.7119	0.6570	0.3982	0.3982	**0.7344**	0.7057
*Ionosphere*	351	33	2	0.7024	0.6991	**0.7076**	0.6872	0.3992	0.5004	0.6904	0.7024
*mammographic mass*	830	5	2	0.6774	**0.8137**	0.8067	0.8010	0.5218	0.5374	0.7976	0.7987
*breast cancer wisconsin diagnostic*	569	30	2	0.8268	**0.9370**	0.9181	0.9276	0.4007	0.8832	0.8552	0.9303
*jain*	373	2	2	0.7660	0.8369	**1.0000**	0.7974	0.9127	0.8416	0.9001	0.8636
*flame*	240	2	2	0.8331	0.8461	0.8962	0.8620	0.7986	0.8584	**1.0000**	0.8303
*pathbased*	300	2	3	0.7081	0.7270	0.7586	0.7147	0.7223	**0.7668**	0.7273	0.7270
*iris*	150	4	3	0.8918	0.8593	0.8841	0.8867	0.8841	0.8512	**0.8996**	0.8853
*seeds*	210	7	3	0.8954	0.9104	0.9290	0.9054	0.8795	0.9037	0.9286	**0.9479**
*wine*	178	13	3	0.7032	0.9270	0.9500	0.9425	0.5500	0.8245	0.7860	**0.9722**

Bold: The bold means the first place result of all methods compared.

And NNC performed significantly better (Wilcoxon Rank Sum test’s p value < 0.05, Supplemental Materials 3) than k-means, PAM, CLARA, AGNES and DIANA in F-score on benchmarked 15 datasets. Thus NNC is a competitive method for clustering task.

### Results from psoriasis dataset study

We benchmarked seven methods: k-means, PAM, Hcluster, CLARA, AGNES, DIANA, NNC (Clusterdp was not included was because the psoriasis data was too large, so it took too long to adjust the parameters) in the psoriasis dataset study.

**Figure 1:**
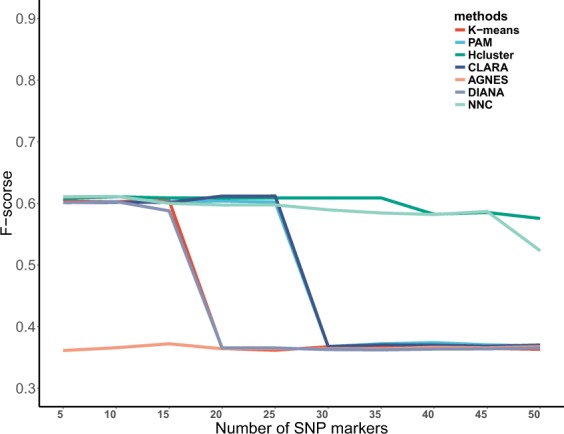
The macro-averaged F-score of selected top 50 associated SNPs on the Psoriasis GWAS dataset of GRU group.

**Figure 2:**
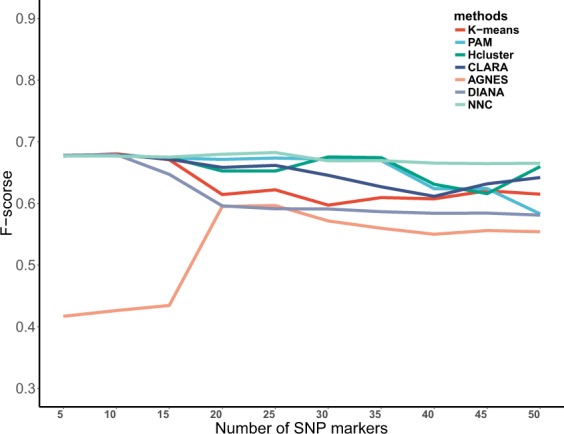
The macro-averaged F-score of selected top 50 associated SNPs on the Psoriasis GWAS dataset of ADO group.

Table 3 presents the mean and standard deviation of each method’s performance among 2 psoriasis GWAS datasets. The macro-averaged F-score of selected 50 top associated SNPs (take 5 intervals) were shown in Supplemental Materials 4. In Table 3, we observed that NNC had the second largest mean of F-score (mean = 0.5735) in psoriasis dataset of GRU group and the maximal mean of F-score (mean = 0.6725) in psoriasis dataset of ADO group, and the mean differences between NNC and the next best performing method were 0.0860 and 0.0135. Additionally, in psoriasis dataset of GRU group, NNC obviously improved the F-score in the benchmarked datasets (improved clustering accuracy = 18%), compared with the third best performing method. While compared to the best performing method, the clustering accuracy of NNC was reduced by 5%. In psoriasis dataset of ADO group, the clustering accuracy of NNC was improved by 2% compared to the second best performing method. And the macro-averaged F-score curves of seven methods on psoriasis dataset 1 and psoriasis dataset 2 were shown in Figs 1 and 2, respectively. More interestingly, we found that the F-score of NNC and Hcluster in the top 50 SNPs were superior to other methods in Fig. 1. In Fig. 2, the F-score of NNC was optimal. In conclusion, NCC performed well in two psoriasis datasets and appears to be superior to its competitor methods: k-means, PAM, Hcluster, CLARA, AGNES and DIANA. It is worth mentioning that NNC appeared to be more robust and less sensitive to potential outliers. Although the F-score of NNC was not the best for all datasets, it was the top performer in both the public and the psoriasis datasets.

**Table 3:** Mean and SD of F-score on 2 psoriasis datasets.

methods	Psoriasis 1			Psoriasis 2		
	Mean 1	SD	Pvalue	Mean 2	SD	Pvalue
k-means	0.4363	0.1155	**1.9531E-03**	0.6314	0.0316	**2.9297E-03**
PAM	0.4864	0.1221	**2.4414E-02**	0.6548	0.0328	6.5430E-02
Hcluster	**0.6006**	0.0138	9.8145E-01	0.6590	0.0214	5.3664E-02
CLARA	0.4875	0.1247	9.6680E-02	0.6507	0.0229	**4.8828E-03**
AGNES	0.3654	0.0029	**9.7656E-04**	0.5261	0.0711	**9.7656E-04**
DIANA	0.4340	0.1127	**9.7656E-04**	0.6119	0.0401	**4.8828E-03**
NNC	0.5735	0.0722	—	**0.6725**	0.0065	—

Bold: The bold means the first place result of all methods compared. SD: Standard Deviation.

The pvalue was calculated by Wilcoxon Rank Sum test (paired = TRUE, alternative = “greater”).

## Discussion

Clustering has been applied for identifying groups among the observations^4^. For example, using clustering to classify patients into subgroups according to their gene expression profile in cancer research. It can be useful for identifying the molecular profile of patients with good or bad prognostic, as well as for understanding the disease.

NNC outperforms the k-means clustering by breaking its limitations: K-means attempts to minimize the total squared error, which is sensitive to the outliers. Furthermore, k-means performed not well in datasets (like *Indian liver patient* and *Parkinsons*, Table 2) with strong correlation coefficient matrixes. To overcome the limitation, we employed the nuclear norm as a measure of clustering fitness. First, nuclear norm^40^ is a L1 measure of error, thus is relatively more robust than squared error. Second, in the presence of variable correlation, nuclear norm internally orthogonalizes the variables and penalizes/down-weights correlated variables.

NNC, along with Clusterdp and Hcluster, had the best performance in more public datasets (Table 2). And we found that these three methods performed best on different public datasets. They could be complementary methods in different real datasets. Furthermore, we observed that the datasets in which NNC performed better were linearly separable (especially in *iris, seeds* and *wine* datasets).

NNC has two parameters, the desired number of cluster K and the number of iterations. The greater the number of iterations, the more precise the convergence. But if the number of iterations is too large, it will affect the computing efficiency. In the psoriasis GWAS datasets, the parameters were chosen as follows: K = 2, the number of iterations = 200000. Generally, when the number of iterations is 20000, NNC also performs well enough (robust with the parameters). The computation complexity of NNC is O(sample number × feature number × min(sample number, feature number) × iterations). When there are 10k or 100k objects in the dataset, it will be rather slow. However, NNC is fast enough (Table 4) to handle medium size datasets (below 10k) in practice.

**Table 4:** The detail running time comparison of all benchmarked methods.

Methods	Psoriasis 1 with 1590 samples (50 SNPs)	Psoriasis 2 with 1133 samples (50 SNPs)
	Computing Time (seconds)	Computing Time (seconds)
k-means^#^	0.030	0.025
PAM^#^	0.053	0.030
Hcluster^#^	0.056	0.025
CLARA^#^	**0.016**	**0.018**
AGNES^#^	0.041	0.035
DIANA^#^	0.084	0.036
NNC(iter = 20, K = 2)	0.016	0.012
NNC(iter = 200, K = 2)	0.053	0.024
NNC(iter = 2000, K = 2)	0.414	0.735
NNC(iter = 20000, K = 2)	4.754	7.328
NNC(iter = 200000, K = 2)	82.757	86.205

Bold: The bold means the first place running time of all methods compared.

Computing time: The time calculated on the processor.

^#^Sum of 10 times computing time according to the default parameters.

In conclusion, we presented the Nuclear Norm Clustering (NNC) method and our work demonstrated that NNC is a rather promising alternative method for clustering in medium size datasets.

## Electronic supplementary material

Supplemental Materials 1

Supplemental Materials 2

Supplemental Materials 3

Supplemental Materials 4
