# Supplementary material for: Nuclear Norm Clustering: a promising alternative method for clustering tasks
Source: Sci Rep. 2018 Jul 18;8:10873. doi: 10.1038/s41598-018-29246-4 (PMC6052164; doi:10.1038/s41598-018-29246-4)
Supplement: Supplementary file 1 — Supplemental Materials 1 [file 41598_2018_29246_MOESM1_ESM.pdf]

Nuclear Norm Clustering: a promising alternative method for clustering tasks

Yi Wang[1]☯, Yi Li[1]☯, Chunhong Qiao[1]☯, Xiaoyu Liu[1]☯, Meng Hao[1]☯, Yin Yao Shugart[2,3*], Momiao Xiong[4*], Li Jin[2*]

[1]Ministry of Education Key Laboratory of Contemporary Anthropology, Collaborative Innovation Center for Genetics and Development, School of Life Sciences and Fudan Institute of Human Phenome, Fudan University, Shanghai, China

[2]State Key Laboratory of Genetic Engineering, Collaborative Innovation Center for Genetics and Development, School of Life Sciences, Fudan University, Shanghai, China.

[3]Unit on Statistical Genomics, Division of Intramural Division Programs, National Institute of Mental Health, National Institutes of Health, Bethesda, MD, USA

[4]Human Genetics Center, School of Public Health, University of Texas Houston Health Sciences Center, Houston, Texas, USA.

☯The five authors contributed equally to the work

***Corresponding authors:**

E-mail:

yinyao21043@gmail.com **(YYS),**

momiao.xiong@gmail.com **(MMX),**

lijin@fudan.edu.cn **(LJ).**

R Code For Clustering

```
library(cluster)
#processing the data
x=read.table("example_x.txt")
x=scale(x)
n=dim(x)[1]
k=3 #Or k=2

#Kmeans
kmeans_result=matrix(0,nrow=n,ncol=10)
for(i in 1:10){
    ns=10*i
    kclust=kmeans(x,k,iter.max=100,nstart=ns)
    yhat=kclust$cluster
    kmeans_result[,i]=yhat
}
write.table(kmeans_result,"kmeans_result.txt",row.names=F,col.names=F,quote=F,sep="\t")

#PAM
pam_result=matrix(0,nrow=n,ncol=2)
metric=c("euclidean","manhattan")
for(i in 1:2){
    m=metric[i]
    p=pam(x,k,metric=m)
    yhat=p$clustering
    pam_result[,i]=yhat
}
write.table(pam_result,"pam_result.txt",row.names=F,col.names=F,quote=F,sep="\t")

#Hcluster
method=c("ward.D", "ward.D2", "single", "complete", "average","mcquitty","median","centroid")
lm=length(method)
distance=c("euclidean", "maximum", "manhattan", "canberra", "binary","minkowski")
ld=length(distance)
hcluster_result=matrix(0,nrow=n,ncol=lm*ld)
for (i in (1:lm))
{
  m=method[i]
  for (j in (1:ld)){
    d=distance[j]
    xdist=dist(x,method=d)
    h=hclust(xdist,method=m)
    k=ld*(i-1)+j
    yhat=cutree(h,k)
    hcluster_result[,k]=yhat
  }
}
write.table(hcluster_result,"hcluster_result.txt",row.names=F,col.names=F,quote=F,sep="\t")

#CLARA
clara_result=matrix(0,nrow=n,ncol=2)
```

```
metric=c("euclidean","manhattan")
for(i in 1:2){
    m=metric[i]
    c=clara(x,k,metric=m)
    yhat=c$clustering
    clara_result[,i]=yhat
}
write.table(clara_result,"clara_result.txt",row.names=F,col.names=F,quote=F,sep="\t")

#AGNES
metric=c("euclidean","manhattan")
lmc=length(metric)
method=c("ward.D", "ward.D2", "single", "complete", "average","mcquitty","median","centroid")
lmd=length(method)

agnes_result=matrix(0,nrow=n,ncol=lmc*lmd)
for(i in 1:lmd){
    md=method[i]
    for(j in 1:lmc){
        me=metric[j]
        a=agnes(x,metric=me)
        yhat=cutree(as.hclust(a,method=md),k)
        z=lmc*(i-1)+j
        agnes_result[,z]=yhat
    }
}
write.table(agnes_result,"agnes_result.txt",row.names=F,col.names=F,quote=F,sep="\t")

#DIANA
metric=c("euclidean","manhattan")
lmc=length(metric)
method=c("ward.D", "ward.D2", "single", "complete", "average","mcquitty","median","centroid")
lmd=length(method)

diana_result=matrix(0,nrow=n,ncol=lmc*lmd)
for(i in 1:lmd){
    md=method[i]
    for(j in 1:lmc){
        me=metric[j]
        d=diana(x,metric=me)
        yhat=cutree(as.hclust(d,method=md),k)
        z=lmc*(i-1)+j
        diana_result[,z]=yhat
    }
}
write.table(diana_result,"diana_result.txt",row.names=F,col.names=F,quote=F,sep="\t")

#Clusterdp
library("densityClust")
Dist=dist(x)

##plot to determine the interval of dc and threshold parameters(delta and rho).
tiff("clusterdp.tiff",width=2400,height=30000)
```

```
par(mfrow=c(100,5))
for(i in 0:499){
  d=i/10
  Clust=densityClust(Dist,dc=d)
  p=paste("dc=",as.character(d),sep="")
  plot(Clust)
  title(main=p,adj=0.3)
}
dev.off()

clusterdp_result=matrix(0,nrow=n,ncol=1000)
for(i in 151:1150){ #interval depends on the above plot
  d=i/100
  Clust=densityClust(Dist,dc=d)
  Clust=findClusters(Clust,delta=6,rho=0,plot=F)
  yhat=Clust$clusters
  z=i-150
  clusterdp_result[,z]=yhat
}

write.table(clusterdp_result,file="clusterdp_result.txt",row.names = F,col.names = F,quote = F,sep='\t'
```
